# HDR Merging of RAW Exposure Series for All-Sky Cameras: A Comparative Study for Circumsolar Radiometry

**DOI:** 10.3390/jimaging11120442

**Published:** 2025-12-11

**Authors:** Paul Matteschk, Max Aragón, Jose Gomez, Jacob K. Thorning, Stefanie Meilinger, Sebastian Houben

**Affiliations:** 1 International Centre for Sustainable Development (IZNE), University of Applied Sciences Bonn-Rhein-Sieg, 53757 Sankt Augustin, Germany; 2 Wematics FlexCo, 5020 Salzburg, Austria; 3 Mines Paris, PSL University, Centre for Observation, Impacts, Energy (O.I.E.), Sophia-Antipolis, 06904 Antibes, France; 4 Solar Photovoltaic Systems, Department of Electrical and Photonics Engineering, Technical University of Denmark (DTU), 4000 Roskilde, Denmark; 5 Institute for Artificial Intelligence and Autonomous Systems (A2S), University of Applied Sciences Bonn-Rhein-Sieg, 53757 Sankt Augustin, Germany

**Keywords:** all-sky imager (ASI), exposure-series fusion, high dynamic range (HDR), circumsolar region, RAW-domain HDR, image signal processing (ISP)

## Abstract

All-sky imagers (ASIs) used in solar energy meteorology face an extreme intra-image dynamic range, with the circumsolar neighborhood orders of magnitude brighter than the diffuse dome. Many operational ASI pipelines address this gap with high-dynamic-range (HDR) bracketing inside the camera’s image signal processor (ISP), i.e., after demosaicing and color processing in a nonlinear 8-bit RGB domain. Near the Sun, such ISP-domain HDR can down-weight the shortest exposure, retain clipped or near-clipped samples from longer frames, and compress highlight contrast, thereby increasing circumsolar saturation and flattening aureole gradients. A radiance-linear HDR fusion in the sensor/RAW domain (RAW–HDR) is therefore contrasted with the vendor ISP-based HDR mode (ISP–HDR). Solar-based geometric calibration enables Sun-centered analysis. Paired, interleaved acquisitions under clear-sky and broken-cloud conditions are evaluated using two circumsolar performance criteria per RGB channel: (i) saturated-area fraction in concentric rings and (ii) a median-based radial gradient in defined arcs. All quantitative analyses operate on the radiance-linear HDR result; post-merge tone mapping is only used for visualization. Across conditions, ISP–HDR exhibits roughly double the near-saturation within 0–$4^{{}^{\circ}}$ of the Sun and about a three- to fourfold weaker circumsolar radial gradient within 0–$6^{{}^{\circ}}$ relative to RAW–HDR. These findings indicate that radiance-linear fusion in the RAW domain better preserves circumsolar structure than the examined ISP-domain HDR mode and thus provides more suitable input for downstream tasks such as cloud–edge detection, aerosol retrieval, and irradiance estimation.

## 1. Introduction

A key challenge for all-sky imagers (ASIs) used in solar energy meteorology is the extreme dynamic range in hemispheric scenes, with orders-of-magnitude differences between the diffuse sky and the circumsolar region. The circumsolar region—defined here as the near-Sun scattered field outside the solar disk (circumsolar aureole)—is critical for resolving cloud transits across the solar disk, which affect direct normal irradiance (DNI), and for characterizing near-Sun scattering in the surrounding aureole. Both mechanisms strongly modulate surface solar irradiance (SSI). Small radiance errors in this area propagate into SSI and its short-term variability. Preserving circumsolar structure with adequate saturation headroom in a radiance-linear sensor domain is therefore important, since this region is highly susceptible to sensor clipping, ISP compression, and halo artifacts [1,2,3,4].

Single-exposure imaging faces a fundamental trade-off. The shortest shutter preserves circumsolar structure but underexposes the sky dome; longer shutters recover the diffuse sky at the cost of larger saturated areas and weaker circumsolar gradients. Highlight headroom is bounded by pixel full-well capacity and, once analog gain is applied, by the converter’s white level. Without optical attenuation (e.g., neutral-density or solar filters), the radiance of the solar disk typically exceeds the single-exposure dynamic range of visible-range CMOS sensors; the disk cannot be resolved and the core saturates in any single capture. In practice, the objective is to minimize the angular footprint of saturation and to preserve circumsolar radial gradients while maintaining adequate signal over the diffuse hemisphere. These constraints motivate exposure bracketing and HDR fusion [5,6,7].

In many operational ASI systems, the dynamic-range gap is addressed with high-dynamic-range (HDR) bracketing inside the image signal processor (ISP), i.e., after demosaicing and color processing in a nonlinear 8-bit RGB domain. Near the Sun, such ISP-domain HDR can lose information via two mechanisms: (i) exposure-series weighting that down-weights the darkest (shortest) frame and can admit clipped or near-clipped samples from longer frames, biasing highlights; and (ii) global or local tone mapping that compresses highlight contrast and can introduce halos, attenuating circumsolar gradients [4,5,6,7]. These mechanisms directly affect the circumsolar region and thus the information content available for irradiance-related and atmospheric applications.

In this context, a radiance-linear HDR fusion in the sensor/RAW domain (RAW–HDR) is contrasted with the vendor ISP-based HDR mode (ISP–HDR) for an all-sky camera system. Solar-based geometric calibration provides a mapping between image coordinates and sky directions and enables Sun-centered analysis. RAW exposure series are merged in a radiance-linear sensor domain by strict per-pixel censoring of saturated, near-saturated, and sub-noise samples, followed by a bounded linear estimate from the usable exposures. A monotone, saturation-count-informed post-merge scaling is used for visualization only; all quantitative analyses operate on the radiance-linear HDR result.

The main contribution of this paper is a quantitative, Sun-centered evaluation of ISP-based HDR versus radiance-linear RAW-based HDR in the circumsolar region of all-sky images. This evaluation is based on an interleaved acquisition scheme that provides paired ISP–HDR frames and RAW exposure series under clear-sky and broken-cloud conditions for a representative all-sky camera system. The RAW series are merged in a documented radiance-linear HDR configuration with fixed analog gain and strict per-pixel censoring of saturated and sub-noise samples. Using a geometric calibration derived from solar observations, a Sun-centered evaluation framework is employed, within which two geometry-aware circumsolar performance metrics are defined: (M1) the saturated-area fraction in concentric rings and (M2) a median-based radial gradient in defined arcs. These metrics are used to quantify differences between RAW–HDR and ISP–HDR. In this framework, ISP–HDR exhibits approximately 1.8–2.2× higher near-saturation within 0–$4^{{}^{\circ}}$ and 3.2–4.6× weaker circumsolar radial gradients within 0–$6^{{}^{\circ}}$ relative to RAW–HDR.

## 2. All-Sky Camera and Data Acquisition

This study uses data from a single node of the Wematics *PyranoVision* network installed at the Technical University of Denmark (DTU). Denmark has a temperate oceanic climate (Köppen Cfb) with mild summers, high humidity, frequent cloud cover, and persistent winds from the North Sea and Baltic. This section summarizes the capture stack and the acquisition protocol used for the paired HDR comparison.

### 2.1. System Overview

**Figure 1:**
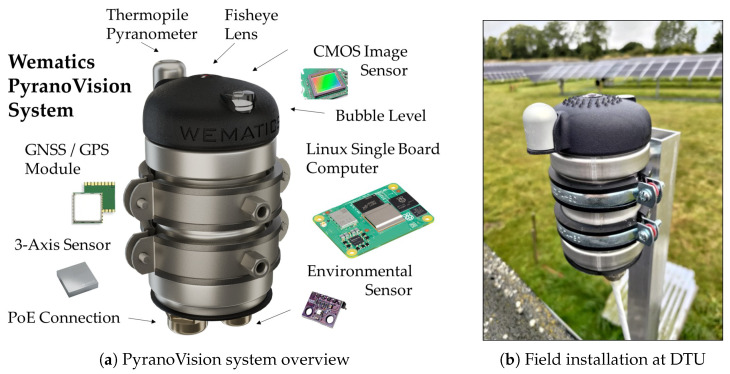
PyranoVision hardware: (**a**) 3D-rendered overview of major components; (**b**) field installation at DTU.

The *PyranoVision* system integrates a weather-sealed fisheye sky camera (12 MP, IR-cut filter) and a thermopile pyranometer for global horizontal irradiance (GHI) on a shared single-board computer (SBC). The SBC performs continuous acquisition, lightweight background compression, and metadata logging. The pyranometer is an ISO 9060:2018 Class C instrument [8], calibrated against an international standard with traceability to the World Radiometric Reference (WRR) via an ISO/IEC 17025-accredited laboratory [9]. Both sensors are controlled by the same acquisition process and synchronized to a common system clock [10], yielding co-registered image brackets and irradiance measurements with identical timestamps. Figure 1 situates the hardware: panel (a) sketches the major components; panel (b) shows the DTU field installation.

For completeness, key hardware characteristics are summarized in Table 1.

### 2.2. Capture Stack and Modes

Data acquisition was implemented in Python 3.11. A single control script provides two operating modes:

1. **RAW–HDR mode** (sensor/RAW domain): multi-exposure brackets of 12-bit Bayer frames are captured via Raspberry Pi’s Picamera2 library (v0.3.13) [11], which is built on libcamera (v0.2.0) [12,13]. Each bracket follows predefined exposure times; eight frames are saved losslessly with per-frame metadata.
2. **ISP–HDR mode** (camera/ISP domain): a single 8-bit, post-demosaicing HDR image is produced by invoking the vendor pipeline from Python via libcamera-still (v1.4) [14]; multi-frame combination and tone mapping follow the vendor defaults.

Both modes are configured from the same Python script. For RAW–HDR, exposure time and analog gain are set through PiCamera2/libcamera controls [11,13]; for ISP–HDR, parameters are passed to libcamera-still [14]. All frames carry request start/end timestamps and metadata (exposure, analog gain, mode flags).

### 2.3. Field Campaign

The field campaign spanned June–July 2025 (Denmark). Acquisition proceeded at four HDR events per minute with a fixed 15 s alternation to enable paired comparisons: ISP–HDR at {0, 30} s via libcamera-still, and RAW–HDR at {15, 45} s via PiCamera2. For RAW–HDR, automatic exposure and automatic white balance were disabled, analog gain was fixed, and a predetermined exposure series was executed for each bracket (see Section 4.2). Timestamps were recorded in ISO 8601 with explicit offset and stored alongside per-frame metadata, consistent with solar-resource data guidelines [15].

## 3. Geometric Calibration

Geometric calibration defines a mapping from sky directions to image pixels by combining a fisheye projection model with the camera’s external orientation relative to the horizontal plane and true north. Each pixel is assigned an azimuth (clockwise from true north) and a zenith angle (from the local vertical). Although calibration can be derived from multiple celestial observations (cf. [16]), solar-only methods have also been demonstrated [17]. Clear-sky images from 13 to 14 June 2025 were processed with the Sun-based mode of *SuMo* [18]. Model parameters (intrinsics: projection and principal point; extrinsics: three-axis orientation) were estimated by minimizing the discrepancy between detected solar centroids and solar ephemeris directions across time using a robust objective. Physical calibration targets (e.g., checkerboards) were not required. For downstream analyses, the Sun center at each timestamp was obtained by forward-projecting the ephemeris direction through the calibrated model. This provides a precise, temporally continuous reference that is independent of image-based detection (cf. [19]) and ensures a consistent definition of circumsolar regions for precise Sun-crop extraction.

### 3.1. Calibration Procedure

**Figure 2:**
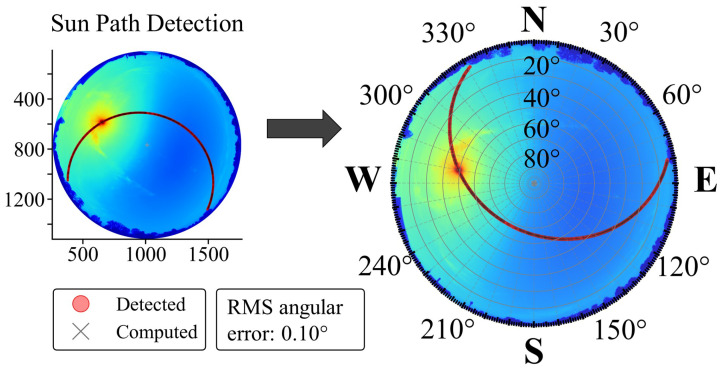
Sun-path detection and geometric calibration. Left: detected (red) and computed (black) solar path in image coordinates. Right: radar-style overlay (clockwise azimuth, north up) assigning azimuth and zenith to sky pixels. Background image: 2025-06-13T16:47:45+02:00, shown with a jet colormap. Sample size N=2314. Dataset (UTC+2): 13 June 2025, 05:16–20:57, and 14 June 2025, 05:15–20:59.

Solar centroids were extracted from clear-sky daytime images on 13–14 June 2025 (Figure 2). A fixed sky mask was applied, and the lowest-exposure frame per time was used to identify the Sun. Within a local window around the predicted Sun neighborhood, intensity thresholding with minor morphological cleanup was performed. Candidates were screened by circularity *C* and a minimum area $A_{\min}$ to ensure a well-defined Sun-disk region. Images in which the Sun disk intersected the mask boundary were rejected, and the intensity-weighted centroid of the best candidate was retained. Temporal consistency was enforced by fitting an elliptical Sun path in image coordinates and discarding points with orthogonal deviation >1 px. Accepted timestamps $t_{i}$ were paired with ephemeris directions (Section 3.2). Table 2 summarizes the symbols used in geometric calibration.

For observation *i*, the model maps the centroid to $v_{i}^{\mathrm{obs}}\left(\Theta \right)$, and ephemerides provide $v_{i}^{\mathrm{eph}}$. The loss minimized the great-circle misalignment via the squared Euclidean norm

(1) $$\min_{\Theta }\;\frac{1}{N}\sum_{i=1}^{N}{∥v_{i}^{\mathrm{obs}}\left(\Theta \right)\times v_{i}^{\mathrm{eph}}∥}^{2},$$

with an optional Huber penalty and per-sample quality weights. Following Blum et al. [18], a coarse-to-fine scheme was adopted: the principal point c and the global orientation were initialized, after which both the fisheye projection (intrinsics) and the orientation (extrinsics) were jointly refined.

The June dataset spans a broad range of azimuth and zenith angles. As shown in Figure 2, the solar track covers azimuths of approximately 80–$330^{{}^{\circ}}$ (sunrise near the east, sunset toward the northwest) and zenith angles of roughly 7–$60^{{}^{\circ}}$.

Residuals are reported in degrees and are summarized by distribution quantiles and polar residual maps. For N=2314 detections, the observed–ephemeris angular residuals are small, with a root-mean-square angular error of $0.10^{{}^{\circ}}$.

### 3.2. Solar Position Mapping and Calibration Outputs

Solar unit vectors were computed from standard ephemerides using image timestamps; no refraction correction was applied. The calibrated inverse map (u,v)↦(θ,ϕ) (zenith angle θ, azimuth ϕ clockwise from north) enables zenith-angle binning and reproducible, geometry-defined circumsolar regions around the ephemeris Sun direction.

The archived calibration products comprise the image center c, the sky–circle radius *R*, the intrinsic fisheye projection, and the extrinsic orientation with respect to the local horizon and north; these suffice to project the ephemeris Sun direction into image space and to map pixels to (θ,ϕ) for all subsequent analyses.

## 4. HDR Merging of RAW Exposure Series

This section describes the HDR processing used in this study. A radiance-linear HDR fusion in the sensor/RAW domain is compared with an operational ISP-domain HDR mode. Three radiometric quantities are used: radiance (per-pixel directional quantity, W/m^2^/sr), irradiance (pyranometer plane-integrated flux, W/m^2^, used only as an external reference), and digital numbers (DNs; raw sensor counts). For fixed gain and exposure time, non-saturated DNs are treated as proportional to radiance, so HDR fusion operates on DNs as a radiance-linear proxy. The RAW-domain pipeline follows established HDR approaches [5,6,7] and previous HDR applications to all-sky imaging [20].

### 4.1. ISP-HDR Pipeline

The ISP-based HDR reference was obtained via the libcamera command-line utility with the vendor HDR option enabled. Processing is performed after Bayer demosaicing and color processing in a nonlinear 8-bit RGB domain. Multiple frames acquired at the same exposure are internally accumulated mainly for noise suppression; the minimum exposure time within the bracket is configured to limit highlight clipping. A global tone-mapping stage and a mild local-contrast enhancement determine the displayed appearance. This ISP–HDR mode represents the operational configuration in Wematics all-sky cameras; the RAW-domain fusion described below is used for quantitative evaluation.

### 4.2. RAW-HDR Pipeline

The RAW–HDR pipeline merges exposure series directly in the 12-bit Bayer domain under fixed analog gain, with auto exposure (AE) and auto white balance (AWB) disabled. At each timestamp, K=8 RAW frames (RGGB, 2028×1520 px) are captured with measured exposure times

$$t_{\mu }=\left\{60,\;136,\;273,\;591,\;985,\;1986,\;3988,\;7992\right\}\;\mu s,$$

with deviations from the commanded values below ±3%. The series spans from the shortest achievable exposure to clearly saturated images, providing overlap for HDR fusion. The sensor operates with a global black level B=0 DN and a 12-bit clip level $C_{\mathrm{clip}}=4095$ DN.

For each exposure *k* and pixel (i,j), raw counts $x_{k}\left(i,j\right)$ are black-level-corrected and clipped to obtain

(2) $$y_{k}\left(i,j\right)=\mathrm{clip}\;\left(x_{k}\left(i,j\right)-B,\;0,\;C_{\mathrm{clip}}\right).$$

In the non-saturated regime and for fixed gain, these measurements are assumed to follow a simple exposure–response relation

(3) $$y_{k}\left(i,j\right)\approx r\left(i,j\right)\;t_{k},$$

where $t_{k}$ is the measured exposure time and r(i,j) denotes the radiometric slope (DN ${\mu s}^{-1}$) at pixel (i,j).

To suppress unusable samples, each exposure at (i,j) is classified according to its DN. Values below a noise threshold $\Delta_{\mathrm{noise}}=8$ DN are discarded as too dark, and values above $C_{\mathrm{clip}}-\Delta_{\mathrm{sat}}$ with $\Delta_{\mathrm{sat}}=32$ DN are discarded as near-saturated. The remaining exposures at that pixel are treated as usable. If at least two usable samples exist, the radiance rate r(i,j) is estimated as the slope of *y* versus *t* using a least-squares fit constrained by the observed range: the fitted slope is restricted to lie between a lower bound implied by near-saturated samples and an upper bound implied by the usable samples (between the smallest slope that would have produced near-saturation and the largest slope consistent with the usable DN values).

If no exposure passes the usability criteria at a given pixel, a simple fallback is applied based on the brightest frame. When all values remain below $y_{\mathrm{dark}}=64$ DN, the slope is set to the value implied by the longest exposure. Otherwise, the pixel is treated as saturated and assigned a conservative minimum slope derived from near-saturated samples at the longest exposures. In all other cases, the bounded least-squares slope is taken as the HDR estimate r(i,j).

**Figure 3:**
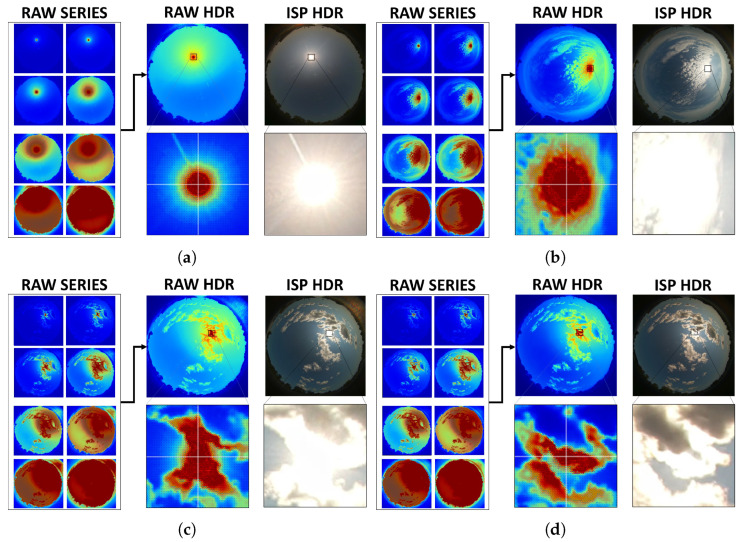
Representative scenes for the RAW–HDR vs ISP–HDR comparison. RAW–HDR merges eight 12-bit RGGB frames (2028×1520 px) in a radiance-linear sensor domain; full frames and 120×120 px Sun-centered crops share a fixed linear intensity scale (no gamma). ISP–HDR shows the vendor RGB output recorded +15 s after each RAW bracket. (**a**) clear sky, (**b**) altocumulus/stratocumulus, (**c**,**d**) cumulus with transient partial solar obstruction; corresponding GHI = 827, 619, 916, and 883 W/m^2^.

For visualization, the radiance-linear HDR field r(i,j) is converted to display RGB using a monotone tone curve with a mild banded rescaling that preserves headroom in the circumsolar region while enhancing contrast in the diffuse dome. All quantitative evaluations in Section 5 operate on the radiance-linear HDR estimate r(i,j). Representative clear-sky and broken-cloud scenes are shown in Figure 3.

Qualitatively, Figure 3 already suggests that RAW–HDR preserves finer circumsolar gradients and exhibits a smaller saturated footprint than ISP–HDR. Section 5 introduces Sun-centered metrics that are used to quantify these differences.

## 5. Circumsolar Metrics

The circumsolar region is characterized by two ring-based, rotation-symmetric metrics: (i) the saturated-area fraction (highlight headroom) and (ii) the median-based radial gradient (aureole fall-off). Rings are defined by the solar elongation ψ (deg), i.e., the great-circle angle between a pixel’s view direction and the Sun. Throughout this study, rings are spaced by $\Delta \psi =0.25^{{}^{\circ}}$ with edges $r_{k}:=k\;\Delta \psi$, $k\in N_{0}$. Table 3 summarizes the notation used for these circumsolar metrics.

For a given timestamp, channel, and processing stage, each pixel (u,v) is assigned a solar elongation ψ(u,v) from the geometric calibration (Section 3). Rings $\left[r_{1},r_{2}\right)$ are then defined by selecting all valid pixels with $r_{1}\leq \psi \left(u,v\right)<r_{2}$. An *adaptive* near-white threshold $\tau_{\mathrm{sat}}$ is estimated per timestamp, ring, and channel and is applied identically to RAW and ISP.

### 5.1. Saturated-Area Fraction

The saturated-area fraction $f_{\mathrm{sat}}$ quantifies the fraction of pixels in a ring that are clipped or near-clipped. For a ring $\left[r_{1},r_{2}\right)$, it is defined as

(4) $$f_{\mathrm{sat}}\left(\left[r_{1},r_{2}\right)\right)=\frac{N_{\mathrm{sat}}\left(\left[r_{1},r_{2}\right)\right)}{N_{\mathrm{pix}}\left(\left[r_{1},r_{2}\right)\right)}\;,$$

where $N_{\mathrm{sat}}\left(\left[r_{1},r_{2}\right)\right)$ denotes the number of pixels in the ring with $I_{c}\left(u,v;\mathrm{stage}\right)\geq \tau_{\mathrm{sat}}$ and $N_{\mathrm{pix}}\left(\left[r_{1},r_{2}\right)\right)$ denotes the total number of valid pixels in that ring (after masking). Larger values of $f_{\mathrm{sat}}$ indicate a larger angular footprint of saturation and reduced highlight headroom. In the subsequent analysis, $f_{\mathrm{sat}}$ is summarized over 0–$4^{{}^{\circ}}$.

### 5.2. Median-Based Radial Gradient

To characterize the aureole fall-off, a median-based radial profile is formed from non-saturated pixels and then differentiated with respect to ψ. First, ring medians $m_{k}$ are defined by

(5) $$m_{k}=\mathrm{median}\left\{\;I_{c}\left(u,v;\mathrm{stage}\right)\;:\;r_{k}\leq \psi \left(u,v\right)<r_{k+1},\;I_{c}\left(u,v;\mathrm{stage}\right)<\tau_{\mathrm{sat}}\right\}.$$

Thus, pixels at or above the near-saturation threshold contribute to $f_{\mathrm{sat}}$ in (4) but are excluded from the medians $m_{k}$. The radial gradient is then approximated by a central finite difference of these medians:

(6) $$g\left(\psi_{k+\frac{1}{2}}\right):=\frac{m_{k+1}-m_{k-1}}{\psi_{k+1}-\psi_{k-1}},\;\psi_{k+\frac{1}{2}}:=\frac{1}{2}\;\left(r_{k}+r_{k+1}\right)\;.$$

Negative values correspond to decreasing brightness with increasing solar elongation, and more negative values indicate a steeper circumsolar aureole. In the subsequent analysis, this gradient is evaluated over 0–$6^{{}^{\circ}}$ using the radiance-linear HDR estimates as input.

In physical terms, $f_{\mathrm{sat}}$ quantifies how much of the inner circumsolar region is effectively unusable because it is clipped or near-clipped, and therefore it measures the available highlight headroom around the solar disk. The gradient describes how fast brightness decays with solar elongation and thus captures the shape of the circumsolar aureole. Together, these two metrics describe complementary aspects of circumsolar radiometry: where information is lost to saturation and how well the remaining aureole structure is preserved.

## 6. Results: Circumsolar Metric Comparison

**Figure 4:**
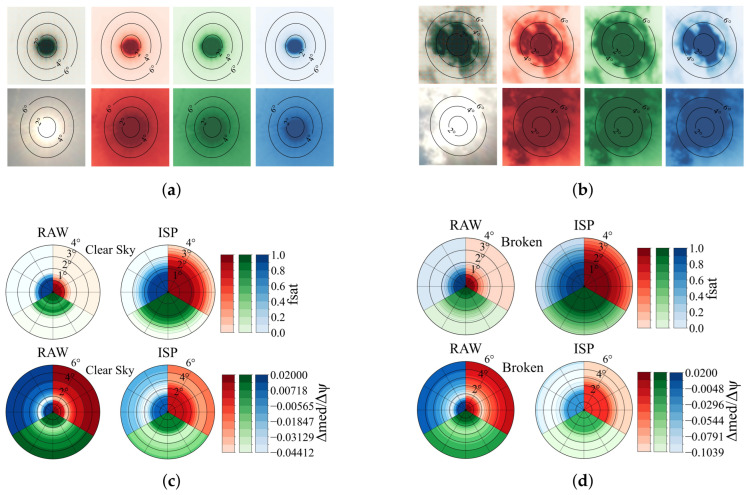
Sun-centered crops and polar aggregates comparing RAW–HDR and ISP–HDR. (**a**,**b**) Example 6° Sun-centered crops for clear-sky and broken-cloud cases (R/G/B). (**c**,**d**) Polar re-projections of ring-wise daily medians (clear vs. broken cloud), averaged across days, with common per-metric color scales; standalone colorbars are shown separately. Scenes with solar elevation $<10^{{}^{\circ}}$ are excluded. In (**c**,**d**), left/right panels show RAW vs. ISP, with (top) circumsolar saturation fraction $f_{\mathrm{sat}}$ (0–4°) and (bottom) radial gradient metric (0–6°) as defined in Section 5.

In this section, results derived from the metrics defined in Section 5 are presented. RAW frames are split into $\left(R,G_{1},G_{2},B\right)$ at half resolution, with $G=(G_{1}+G_{2})/2$. Throughout, $f_{\mathrm{sat}}$ is evaluated over 0–$4^{{}^{\circ}}$ and the radial gradient Δmedian/Δψ over 0–$6^{{}^{\circ}}$; scenes with solar elevation $<10^{{}^{\circ}}$ are excluded. Aggregation uses ring-wise daily medians followed by means across days. Figure 4 summarizes the results.

Panels (a) and (b) show representative Sun-centered crops (6°) contrasting RAW–HDR and ISP–HDR across the R/G/B channels. Panels (c) and (d) show polar aggregates (saturated-area fraction over 0–4° and radial gradient over 0–6°) with common per-metric color scales. The datasets comprise four clear-sky days (89,712 timestamps) and twelve broken-cloud days (158,472 timestamps).

In the RAW domain, inner-ring saturation consistently follows G≳B>R. After ISP processing, this ordering reverses to R>G>B: red becomes most prone to near-clip, green is intermediate, and blue remains least affected. Broken-cloud conditions increase saturation in all channels but do not change these orderings. For the radial fall-off (Δmedian/Δψ), RAW is steeper (more negative) in every channel, whereas ISP uniformly flattens the profile; blue tends to retain the steepest residual decline, red the flattest, with green in between. The corresponding RGB-mean effects are summarized in Table 4.

Overall, RAW–HDR shows about a factor of two lower inner-ring saturation in 0–$4^{{}^{\circ}}$ compared with ISP–HDR, with the reduction slightly stronger under broken cloud (Table 4). Over 0–$6^{{}^{\circ}}$, RAW–HDR also preserves stronger circumsolar gradients: the magnitude |Δmedian/Δψ| is roughly three-quarters larger than in ISP–HDR (equivalently, ISP flattens the profile by approximately 75%). In summary, RAW–HDR better maintains circumsolar headroom and gradient fidelity than the operational ISP mode for the system studied here.

## 7. Discussion

The results show that, for the system studied here, radiance-linear RAW–HDR consistently reduces inner-ring saturation and preserves steeper circumsolar gradients than ISP–HDR. In the Sun-centered framework of Section 5, ISP–HDR exhibits a higher saturated-area fraction $f_{\mathrm{sat}}\left(0–4^{{}^{\circ}}\right)$ and a flatter median-based radial profile within $0–6^{{}^{\circ}}$ for both clear-sky and broken-cloud conditions.

### 7.1. Interpretation of the Circumsolar Metrics

The saturated-area fraction $f_{\mathrm{sat}}$ in the inner rings quantifies highlight headroom around the solar disk. Larger values of $f_{\mathrm{sat}}\left(0–4^{{}^{\circ}}\right)$ indicate that a greater angular region is clipped or near-clipped and therefore cannot encode radiance variations. Information on cloud edges and aureole structure is then lost in this region.

The median-based radial gradient Δmedian/Δψ characterizes the steepness of the aureole fall-off as a function of solar elongation. Steeper (more negative) gradients correspond to a better-resolved circumsolar aureole, whereas flatter gradients indicate loss of contrast due to clipping or nonlinear compression. Together, $f_{\mathrm{sat}}$ and the radial gradient quantify complementary aspects of circumsolar radiometry (headroom and shape) and both have a direct physical interpretation.

### 7.2. Mechanisms Behind RAW–HDR and ISP–HDR Differences

In the RAW domain, the relative responses of the red, green, and blue channels are governed by the sensor’s spectral quantum efficiency, the Bayer color-filter array, and the IR-cut filter. For the camera used here, this combination yields the highest effective response in green, a similar or slightly lower response in blue, and the lowest response in red. With fixed analog gain and disabled automatic exposure and white balance, this leads to the observed saturation ordering G≳B>R for RAW–HDR in the inner rings.

In the ISP domain, processing is optimized for perceptual appearance. After demosaicing, white balance applies per-channel gains to achieve a neutral gray axis, and a color-correction matrix maps camera RGB to the output space. Because RAW responses are typically green-dominant, daylight scenes often require stronger scaling of red than of green and blue, reducing headroom in the red channel. Subsequent tone mapping then operates on these up-gained channels. In the circumsolar region, where radiance is high and the spectrum is relatively warm, the red channel reaches the tone-curve knee or clip first. This explains the shift in saturation ordering to R>G>B for ISP–HDR and the increased red contribution to $f_{\mathrm{sat}}\left(0–4^{{}^{\circ}}\right)$, as well as the flatter aureole gradients.

RAW–HDR, by contrast, merges exposure-normalized frames in a linear sensor domain and applies any compression as an explicit monotone tone curve after fusion. This avoids combining per-channel white-balance scaling and tone mapping with multi-exposure fusion, and thereby preserves more circumsolar headroom and gradient information for the system studied here.

### 7.3. Implications and Limitations

The observed differences are directly relevant for several ASI-based applications. A smaller saturated footprint and steeper circumsolar gradients increase the number of usable pixels near the solar disk, which is favorable for cloud–edge detection and segmentation. Methods for image-based irradiance retrieval (GHI, DHI, DNI, POA/GTI) rely on the structure of the circumsolar aureole when partitioning direct and diffuse components; a better-resolved aureole is expected to stabilize DNI estimates and partial-occlusion states. Likewise, aerosol-related inversions that exploit near-Sun radiance can benefit from preserved gradients within a few degrees of the solar disk. The present work does not implement new retrieval algorithms but provides a quantified characterization of the circumsolar radiance field that can be used in such task-level comparisons.

Several limitations should be noted. Temporal pairing between RAW–HDR and ISP–HDR frames is imperfect because brackets were alternated at 15 s and matched within ±120 s; aggregation via ring-wise daily medians and across-day means reduces, but does not eliminate, the influence of sub-minute cloud evolution. The analysis is based on a single all-sky system (sensor, optics, ISP tuning), so quantitative values of $f_{\mathrm{sat}}$ and the radial gradient will differ for other hardware, filters, and ISP parameters, even though the evaluation framework itself is generic. Computational cost and storage requirements are higher for RAW–HDR than for ISP–HDR, and these practical trade-offs were not quantified here. Finally, radiance linearity is understood in a sensor-domain sense: pixel codes are treated as proportional to scene radiance within the in-band response of the silicon CMOS sensor under fixed analog gain, without implying absolute spectroradiometry or cross-device equivalence.

## 8. Conclusions

This work compared exposure-series HDR in a radiance-linear sensor domain (RAW–HDR) with an operational ISP-based HDR mode (ISP–HDR) for an all-sky camera in the circumsolar region, using a Sun-centered, geometry-aware framework based on inner-ring saturation and aureole gradients.

For the investigated system, RAW–HDR provided systematically lower inner-ring saturation and steeper circumsolar gradients than ISP–HDR across both clear-sky and broken-cloud conditions. The circumsolar neighborhood around the solar disk remained usable over a larger angular range and retained a sharper aureole fall-off when exposure fusion was performed in the radiance-linear RAW domain.

A large share of existing all-sky imaging studies relies on embedded vendor HDR pipelines in the ISP domain, for example in widely used commercial all-sky cameras such as Mobotix-based systems. The results presented here show that, for the configuration studied, such ISP-domain processing can substantially reduce circumsolar headroom compared with radiance-linear RAW merging. The intention of this work is to make this loss of circumsolar detail explicit and to highlight the potential gains that can be obtained by operating in a radiance-linear RAW domain when circumsolar radiometry is a priority.

The Sun-centered, ring-based metrics introduced in this study provide a compact and reproducible way to characterize circumsolar headroom and gradient preservation across different cameras and HDR configurations, independently of any specific downstream algorithm. In this sense, the study quantifies the differences between RAW–HDR and ISP–HDR for a specific all-sky system under realistic sky conditions and introduces a geometry-aware evaluation protocol that can be reused when documenting HDR strategies in future all-sky imaging deployments.

## 9. Outlook

The present results suggest that it is worthwhile to test radiance-linear RAW–HDR inputs explicitly in downstream applications rather than simply accepting circumsolar information loss.

A first line of work is irradiance retrieval from all-sky radiance maps, including global horizontal (GHI), diffuse horizontal (DHI), direct normal (DNI), and plane-of-array/global tilted irradiance (POA/GTI). Methods such as the PyranoCam approach [21] and related vision-based models [22,23,24,25,26,27,28,29] provide a framework in which RAW–HDR and ISP–HDR inputs can be compared in terms of bias and error for different sky conditions and solar geometries.

A second line of work is short-horizon nowcasting of ramp events under broken cloud [30,31,32,33,34,35,36,37,38,39,40]. Here, the impact of RAW-domain HDR on the timing and magnitude of Sun–cloud occlusions can be quantified explicitly by re-running existing nowcasting models with alternative HDR configurations and evaluating changes in forecast skill.

Third, cloud detection, segmentation, and classification [20,41,42] can be revisited by training or evaluating algorithms on RAW–HDR versus ISP–HDR inputs, with a focus on performance in the circumsolar neighborhood. Finally, aerosol-related inversions that exploit the near-Sun aureole [43,44,45] provide a setting in which the preserved radial gradients from RAW–HDR can be linked directly to retrieval accuracy.

These task-level comparisons would extend the present, system-specific radiometric assessment by quantifying how circumsolar headroom and gradient preservation translate into changes in downstream performance for representative all-sky imager applications. 

## Figures and Tables

**Table 1:** Hardware characteristics of the Wematics PyranoVision system.

Component	Specification
**Compute Platform**	Single-board computer (SBC), quad-core ARM Cortex-A76 @ 2.4 GHz; Power-over-Ethernet (IEEE 802.3af), 15 W max; IP63 enclosure; operating range –25 to +65 °C, 0–100% RH.
**All-Sky Camera (VIS)**	1/2.3^″^ CMOS, 4056 × 3040 px; ∼210° FOV; spectral range 400–720 nm (IR-blocked); rolling shutter; exposure range 60 µs to 120 s.
**Irradiance Sensor (GHI)**	Thermopile pyranometer, ISO 9060:2018 Class C; spectral range 385–2105 nm; measurement range 0–2000 W m^−2^; repeatability <1%; drift <2%/year.
**Environmental Sensing**	Temperature –40 to 85 °C (±1 °C); pressure 300–1100 hPa (±1 hPa); humidity 0–100% RH (±3%).

**Table 2:** Symbols used in geometric calibration.

Symbol	Definition	Units / Range
(u,v)	Image coordinates (origin at top–left).	px
$c=(u_{0},v_{0})$	Image center (principal point).	px
*R*	Sky-circle radius (visible dome).	px
θ,ϕ	Zenith angle; azimuth (clockwise from true north).	$\theta \in [0^{{}^{\circ}},90^{{}^{\circ}}]$, $\phi \in [0^{{}^{\circ}},360^{{}^{\circ}})$
$t_{i}$	Timestamp of observation *i*.	ISO 8601, UTC
$v_{i}^{\mathrm{eph}}$	Sun unit vector from ephemerides at $t_{i}$.	unitless (∥·∥=1)
$v_{i}^{\mathrm{obs}}$	Unit vector inferred from the centroid via the inverse projection.	unitless (∥·∥=1)
Θ	Calibration parameters.	—
A,P,C	Area, perimeter, circularity $C=4\pi A/P^{2}$.	px^2^, px, [0,1]

**Table 3:** Symbols for circumsolar metrics.

Symbol	Definition	Units
ψ(u,v)	Solar elongation of pixel (u,v) (great-circle angle to the Sun).	deg
Δψ	Ring width (fixed to $0.25^{{}^{\circ}}$).	deg
$r_{k}$	Ring edge kΔψ.	deg
$I_{c}\left(u,v;\mathrm{stage}\right)$	Channel value (c∈{R,G,B}) at processing stage stage∈{RAW–HDR,ISP–HDR}.	DN
$\tau_{\mathrm{sat}}$	Near-saturation threshold used to flag highlight pixels.	DN
$f_{\mathrm{sat}}\left(\left[r_{1},r_{2}\right)\right)$	Saturated-area fraction in ring $\left[r_{1},r_{2}\right)$.	—
$m_{k}$	Median channel value in ring $\left[r_{k},r_{k+1}\right)$ with $I_{c}<\tau_{\mathrm{sat}}$.	DN
$g(\psi_{k+\frac{1}{2}})$	Median-based radial gradient at mid-elongation $\psi_{k+\frac{1}{2}}$.	DN deg^−1^

**Table 4:** RGB -mean effects. Δ% for $f_{\mathrm{sat}}$ is relative to RAW. Flattening denotes the relative reduction of |Δmedian/Δψ| versus RAW over 0–$6^{{}^{\circ}}$.

	$f_{\mathrm{sat}}$ (0–4°)				Radial Gradient (0–6°)		
Phase	RAW	ISP	Δ (abs)	Δ (%)	RAW	ISP	Flatten. (%)
Clear	0.355	0.690	0.335	94.4	−0.135	−0.035	74.1
Broken	0.370	0.764	0.394	106.5	−0.078	−0.019	75.6

## Data Availability

The original contributions presented in this study are included in the article material (https://zenodo.org/records/17909546, accessed on 1 December 2025). Further inquiries can be directed to the corresponding author.

## Abbreviations

AE	Auto exposure
AOD	Aerosol optical depth
ASI	All-sky imager
AWB	Auto white balance
CMOS	Complementary metal–oxide–semiconductor
DHI	Diffuse horizontal irradiance
DN	Digital number (sensor counts)
DNI	Direct normal irradiance
DTU	Technical University of Denmark
FOV	Field of view
GHI	Global horizontal irradiance
GTI	Global tilted irradiance
HDR	High dynamic range
ISP	Image signal processor
ISP–HDR	ISP-domain HDR image (vendor pipeline)
LDR	Low dynamic range (single exposure)
ND	Neutral density (filter)
POA	Plane of array
RAW–HDR	Radiance-linear HDR merge in RAW domain
RGB	Red–green–blue color space
RGGB	Bayer mosaic pattern (R–G–G–B)
SBC	Single-board computer
SSI	Surface solar irradiance
UTC	Coordinated Universal Time
WB	White balance
WRR	World Radiometric Reference
